# Electrically Transduced Gas Sensors Based on Semiconducting Metal Oxide Nanowires

**DOI:** 10.3390/s20236781

**Published:** 2020-11-27

**Authors:** Ying Wang, Li Duan, Zhen Deng, Jianhui Liao

**Affiliations:** 1 Key Laboratory of Luminescence & Optical Information, Ministry of Education, School of Science, Beijing Jiaotong University, Beijing 100044, China; yingw@bjtu.edu.cn; 2 Beijing Key Laboratory of Security and Privacy in Intelligent Transportation, Beijing Jiaotong University, Beijing 100044, China; duanli@bjtu.edu.cn; 3 Key Laboratory for Renewable Energy, Beijing Key Laboratory for New Energy Materials and Devices, Beijing National Laboratory for Condensed Matter Physics, Institute of Physics, Chinese Academy of Sciences, Beijing 100190, China; 4 Key Laboratory for the Physics and Chemistry of Nanodevices, Department of Electronics, Peking University, Beijing 100871, China; Jianhui.Liao@pku.edu.cn

**Keywords:** semiconducting metal oxide, nanowires, gas sensor, sensing mechanism, doped/loaded additive, crystal defect, low-power consumption

## Abstract

Semiconducting metal oxide-based nanowires (SMO-NWs) for gas sensors have been extensively studied for their extraordinary surface-to-volume ratio, high chemical and thermal stabilities, high sensitivity, and unique electronic, photonic and mechanical properties. In addition to improving the sensor response, vast developments have recently focused on the fundamental sensing mechanism, low power consumption, as well as novel applications. Herein, this review provides a state-of-art overview of electrically transduced gas sensors based on SMO-NWs. We first discuss the advanced synthesis and assembly techniques for high-quality SMO-NWs, the detailed sensor architectures, as well as the important gas-sensing performance. Relationships between the NWs structure and gas sensing performance are established by understanding general sensitization models related to size and shape, crystal defect, doped and loaded additive, and contact parameters. Moreover, major strategies for low-power gas sensors are proposed, including integrating NWs into microhotplates, self-heating operation, and designing room-temperature gas sensors. Emerging application areas of SMO-NWs-based gas sensors in disease diagnosis, environmental engineering, safety and security, flexible and wearable technology have also been studied. In the end, some insights into new challenges and future prospects for commercialization are highlighted.

## 1. Introduction

Rapid and real-time detection of toxic, flammable and explosive gases at low concentrations is essential in various areas like environmental protection [1,2,3,4], chemical analysis [5,6], safety and security [7,8,9], diagnosing diseases [10,11]. Each year, gas fires and explosion accidents in kitchens occur all over the world, causing heavy losses to personal property and life safety. Thus, methane sensors are the mandatory equipment in many countries to give alarm signals when the concentration of methane reaches the explosive limit. High-sensitive gas sensors are also required to detect air pollutants like nitrogen oxides (NO_x_) and formaldehyde (HCHO), which are produced abundantly by industrial production and interior decoration. Medical studies have recently shown that breath sensors can be translated into clinical diagnostics by monitoring certain volatile organic compounds (VOCs) because they can act as biomarkers for some chronic diseases [12,13]. It is generally believed that the breath of lung cancer patients contains a large amount of benzene series (toluene, xylene, etc.) [14,15,16], and the breath of diabetes patients contains a higher concentration of acetone (>1 ppm) [17,18]. Gas sensors have become an indispensable part of the modern Internet of things (IoT). According to a recent market analysis report, the global gas sensor market is anticipated to reach USD 4.1 billion by 2027 [19]. Therefore, many sensing technologies including the infrared spectroscopy [20], gas chromatography [5,21], colorimetry [22], surface plasmon resonance [23], surface acoustic transducer [24], cantilever [25], electrochemistry [26,27], and capacitor [28], have been developed for high-performance gas sensors. Compared with the above optical, chemical, acoustic or gravimetric sensors, a class of electrically transduced gas sensors are attractive for their rapid and real-time detection, simplicity, miniaturization, portability, the possibility of continuous monitoring, and compatibility with standard electronic equipment.

There are two important aspects in an electrically transduced gas sensor: the sensor architecture and the sensing material. On one hand, electrical parameters (resistance, current, voltage or capacitance) can be measured with different sensor architectures, including chemiresistor, field-effect transistor (FET), diode, chemical capacitor, and electrochemical device. Of these, a chemiresistor consisting of only two electrodes is the most commonly used, and FET sensor configuration with the current modulated by the extra gate electrode is generally regarded as a good platform to study the gas-sensing mechanism. On the other hand, the sensing material is responsible for interacting with the target gases and transforming the chemical signals into electrical signals. Semiconducting metal oxides (SMO) such as stannic oxide (SnO_2_) [29,30], zinc oxide (ZnO) [31,32], tungsten oxide (WO_3_) [33,34], copper oxide (CuO) [35,36], indium oxide (In_2_O_3_) [37,38], nickel oxide (NiO) [39,40] and titanium oxide (TiO_2_) [41,42], are generally regarded one class of the most promising sensing materials for their chemical stability, simple processing, low cost, and high sensitivity performance.

The rapid development of innovative nanomaterials and technologies has promoted the progress of gas sensors since the end of the 1990s. Compared with bulk SMO and their sputtered thin films, one-dimensional nanowires (NWs) offer many advantages for gas sensors, such as high surface-to-volume ratios, Debye lengths comparable to the target gas molecules for superior sensitivity, excellent thermal stability, relatively low power consumption, and low tendency to form aggregates [43,44,45,46]. Till now, SMO-NWs with various sizes, shapes and compositions can be synthesized by physical deposition and vapor/liquid-phase chemical processes [47,48,49]. Assembling strategies like dip-coating, printing, Langmuir–Blodgett (LB) technique, and template assembly have been developed for the alignment of NWs on various substrates [50,51,52,53,54]. Moreover, site-specific growth and in situ integration of NWs have been studied towards large scale fabrication of commercialized sensor products [35,37,55,56]. Modern synthesis and assembly methods allow unprecedented control of structural properties, and yet, the sensing mechanism is still confusing.

The nature of the fundamental operating principle of SMO gas sensors is described as the surface reaction of target gases with ionsorbed oxygen. Here we take the SnO_2_ NW-based chemiresistor as an example [30,57,58,59]. Under ambient conditions, oxygen molecules in air adsorb on the active sites of SnO_2_ surface and accept electrons from the conductance band of SnO_2_ to form oxygen ions (O^−^, O_2_^−^, or O^2−^). The consumption of electrons in this process leads to a depletion region, the depth of which is known as the Debye length. When the SnO_2_ NW sensor is exposed to a reducing gas like ethanol (C_2_H_5_OH) or carbon monoxide (CO), the reducing molecules react with the surface oxygen ions and release electrons back to SnO_2_ so that the sensor works at a lower resistance state. The Debye length (L) is typically on the order of 2–100 nm [60]. Thus, it is likely that electrons can be exhausted for NWs with small diameter (D), leading to high sensitivity to reducing gases, especially when D is comparable to or less than 2L (D-L model achieved by the Yamazoe group [61]). In contrast, some studies have reported that crystal defects in SMO-NWs can significantly affect the gas-sensing performance, which makes the conventional D-L model invalid [62,63,64,65]. The development of in situ and in operando methods plays a key role in giving detailed sensing mechanisms instead of empirical explanations [64,66,67]. Moreover, based on the significant theories proposed by Yamazoe and Morrison in the 1980s [68,69], Degler et al., has recently provided revised concepts for understanding the fundamental mechanisms of doped and loaded additives on SMO-based gas sensing materials [70]. The gas response of an SMO-NWs sensor can also be attributed to contact effects, including both the intra-NW junctions and the electrical contacts [71,72,73]. Instead of finding a correlating model to explain the experimental findings, we focus on the comprehensive survey of the relationship between the materials’ structure and gas sensing performance.

A number of developments in low-power sensing technologies and novel applications have been extensively explored, which are associated with the battery-driven IoT. One well-known challenge of SMO gas sensors is that they usually work at relatively high temperatures of 200–400 °C [74,75,76,77]. The high power dissipation limits their practical application. Therefore, many studies have attempted to lower the operating power of SMO-NWs gas sensors, the methods of which can be described as thermal isolation, self-heating operation and developing room-temperature sensors [4,58,78,79,80,81,82]. Moreover, recent advances in the fabrication and integration of SMO-NWs offer unique possibilities for their application in on-chip sensor arrays, flexible sensors and even smart textiles [45,83,84,85,86]. These innovations are critical to further progress in IoT-based projects such as smart homes, telemedicine and safe driving.

Although several recent reviews have been published concerning one-dimensional nanostructure-based gas sensors, most of them focused on other NWs like carbon nanotube, metal, silicon, GaN and polymer [87,88,89,90,91,92]. The latest comprehensive review on SMO-NWs-based gas sensors is still lacking considering the quickly evolving technologies in this area. In this review, we intend to describe investigations on SMO-NWs and their electrically transduced gas sensors by highlighting the most significant processes related to synthetic methodology, assemblies, fundamental structure–property correlations, strategies for low power consumption and novel applications.

## 2. SMO-NWs: Synthesis, Assembly and Sensory Device

According to the dominant charge carriers, SMO-NWs can be classified into n-type NWs (like ZnO, SnO_2_, In_2_O_3_, WO_3_ and TiO_2_) and p-type NWs (like CuO and NiO). For n-type SMO-NWs, oxygen adsorption causes an electron depleted surface layer, and for p-type SMO-NWs, it causes a hole accumulated layer. Based on the Boltzmann statistics [93,94], the resistance change in the case of an accumulated layer is much smaller than the one corresponding to the depletion layer. Hole mobilities are often very low in p-type oxides (0.2 cm^2^V^−1^ s^−1^ for NiO) [44]. In contrast, SnO_2_ and ZnO NWs with high electron mobility (160 and 200 cm^2^V^−1^ s^−1^, respectively) are highly sensitive materials [46,70,95]. These are the two reasons why p-type SMO-NWs are less used compared to n-type ones, especially for detecting oxidizing gases. However, p-type SMO-NWs are also important topics in gas sensor research because of their low-temperature dependence, high humidity or thermal stability, and a high potential for p-n junction applications [73,96]. Typically, CuO is one of the key p-type SMO materials that have been extensively investigated for reducing gases like CO [73,97,98,99]. In this section, a discussion is organized to cover the SMO-NWs synthesis, assembly, sensor architecture, and important performance parameters.

### 2.1. Synthesis of SMO-NWs

The controllability of ZnO microstructures was demonstrated as early as 1971, using a vapor-phase method [100]. In the late 1990s, Yang and Lieber reported a series of studies on the laser ablation and vapor–liquid–solid (VLS) growth of crystalline SMO-NWs [101]. Moreover, Li et al., reported ordered ZnO NW arrays fabricated by electrodepositing and oxidizing Zn in porous anodic alumina membranes (AAM), which was later described as the template-assisted growth in liquid [102]. Till now, there are many different methods in synthesizing SMO-NWs. Considering the large volume of research work, we selectively present the most prominent methods that can be classified into the strategies of top–down and bottom–up.

**Figure 1:**
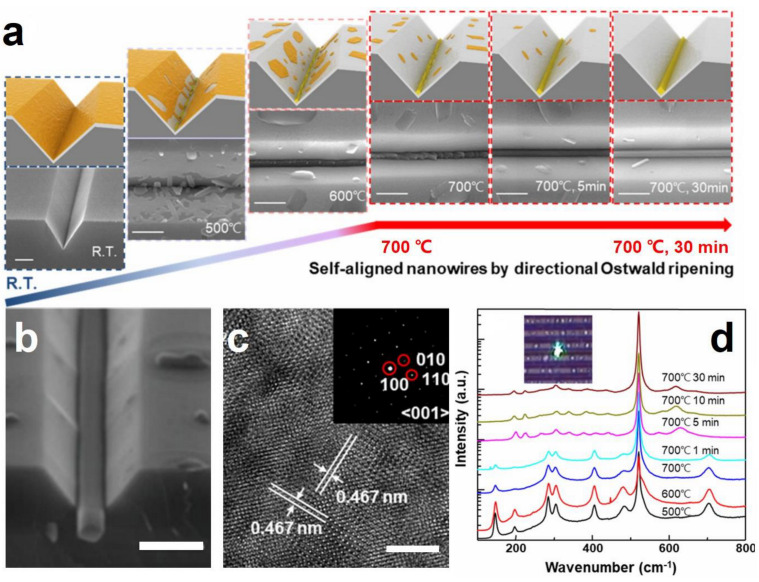
Directional Ostwald ripening for producing aligned arrays of VO_2_ nanowires (NWs). (**a**) Ex situ scanning electron microscope (SEM) images of morphological evolution of the VO_2_ as a function of temperature and growth time. Scale bar: 1 μm. (**b**) SEM images with the angle of the V-groove 70°. Scale bar: 1 μm. (**c**) HR-TEM image of the VO_2_ NW. Scale bar: 5 nm. (**d**) Raman spectra obtained with the laser incident on the V-grooved surface. Reprinted from reference [105] with permission from the American Chemical Society.

The top–down preparation techniques obtain NWs from SMO thin films through combined lithographic methods with physical or chemical depositions [98,103,104,105,106]. In this case, SMO-NWs can be directly incorporated into a sensor circuit without an additional assembly step. The precise position, aligned structure and highly uniform dimensions also benefit the integration of NWs into multifunctional devices. However, the challenge for top–down techniques is that it becomes increasingly difficult to fabricate regular NWs in nanoscale dimensions. For example, Cho et al., prepared a well-aligned PdO NW array using the lithographically patterned Pd NW electrodeposition followed by subsequent thermal oxidation [103]. The PdO NWs are 150 nm in diameter, while the NW edges are irregular and intermittently interconnected with poor electrical conductivity. Using a textured growth substrate can achieve NWs with smaller diameters. Recently, aligned vanadium oxide (VO_2_) NWs with tunable lateral dimensions of 20–690 nm are produced after heating V_2_O_5_ thin films on a grooved silicon oxide (SiO_2_) surface, which is based on a directional Ostwald ripening process (Figure 1a,b) [105]. The high-resolution transmission electron microscope (HR-TEM) image in Figure 1c shows the marked lattice spacing of 0.467 nm corresponding to the interplane spacing of the (010) and (001) planes of monoclinic VO_2_ crystals. The significant changes in Raman spectra with increasing annealing temperature also demonstrate that the V_2_O_5_ is converted to VO_2_ at 700 °C for 30 min (Figure 1d). In contrast, additional transfer of VO_2_ NWs onto other substrates is necessary before fabricating sensor devices. It is also challenging to fabricate single crystalline SMO-NWs by top–down methods.

**Figure 2:**
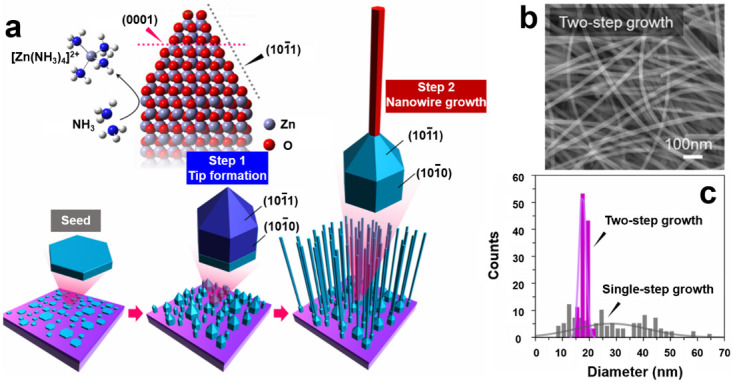
Synthesis of monodispersedly sized ZnO NWs from randomly sized seeds. (**a**) Schematic illustration of the growth process. (**b**) SEM images of fabricated ZnO NWs. (**c**) The narrow diameter distribution of the two-step grown ZnO NWs. Reprinted from reference [120] with permission from the American Chemical Society.

By contrast, the bottom–up techniques assemble atomic or molecular precursors into NWs through a vapor phase or a liquid phase process, which offer several advantages such as flexibility, low cost and simplicity. Vapor-phase synthesis is generally performed inside a horizontal tube furnace under appropriate conditions of source, substrate, catalyst, temperature, pressure and gas flow [101,102,106,107,108,109,110,111,112,113,114]. The growth mechanism can be well defined by vapor–liquid–solid (VLS), vapor–solid (VS), vapor–solid–solid (VSS), oxide-assisted growth or self-catalytic growth [47,107,108]. In recent years, many efforts have been devoted to the rational design for improving the crystal quality, reducing growth temperature of SMO-NWs and growing NWs on amorphous substrates. Takeshi’s group provided the concept of temperature-dependent “material flux window”, and successfully fabricated SMO-NWs like magnesium oxide (MgO), SnO_2_ and ZnO on polyimide substrates at relatively low growth temperatures [112,113]. Recently, Güniat et al., have given impressive insights on the vapor-phase synthesis of NWs [108]. They have summarized the open questions to be explored, such as reducing the size distribution, understanding the microscopic interaction of the NW interface, explaining the defect formation, etc. Correspondingly, liquid-phase synthesis and growth of SMO-NWs are performed in chemical solutions. Typical process variations have been comprehensively discussed in the case of liquid-phase synthesis, including hydrothermal, solvothermal, molten salt, template-assisted, electrodeposition, and electrostatic spinning synthesis of SMO-NWs [57,115,116,117,118,119]. As shown in Figure 2, Zhao et al., have recently succeeded to rationally synthesize monodispersed ZnO NWs from randomly sized seeds by a two-step hydrothermal growth [120]. Figure 2c shows that the distribution of ZnO NW diameters σ is around 1.3 nm; the best result to the authors’ knowledge. This proposed concept is also of generality for other SMO-NWs and thus paves the way for NWs-integrated sensors with reliable performance. Despite the tremendous progress, the main challenge for vapor-phase and liquid-phase synthesis methods is the micro electro mechanical system (MEMS) compatible incorporation of NWs on sensor circuits.

Towards the practical application of SMO-NWs, the integration of top–down and bottom–up strategies is required to combine their merits. The first all-integrated sensor circuit based on cadmium selenide (CdSe) and germanium/silicon (Ge/Si) NWs was reported by the Javey group in 2008, showing the advantages of combining vapor-phase synthesis, printing and photolithography techniques [121]. Moreover, Marasso et al., combined the top–down and bottom–up strategies by developing a polymeric-mask centrifugation method to deposit ZnO nanostructures on MEMS micro-hotplates [122]. Santra et al., reported the mask-less deposition of chemically synthesized Au–SnO_2_ nanocomposites on MEMS platform through the use of dip-pen nanolithography (DPN) to create a low-cost ethanol sensor [75]. Extensive studies have also focused on the local and site-specific growth of SMO-NWs on microhotplates for on-chip integration [35,56,83,84,123,124]. On one hand, in situ electrical and gas sensing measurements can be achieved for revealing new insights into sensor degradation. On the other hand, NW-based electronic noses can be fabricated based on various SMO-NWs materials combining chemical vapor deposition or electrostatic spinning (bottom–up) with lithography technique (top–down).

### 2.2. Assemblies of SMO-NWs

**Figure 3:**
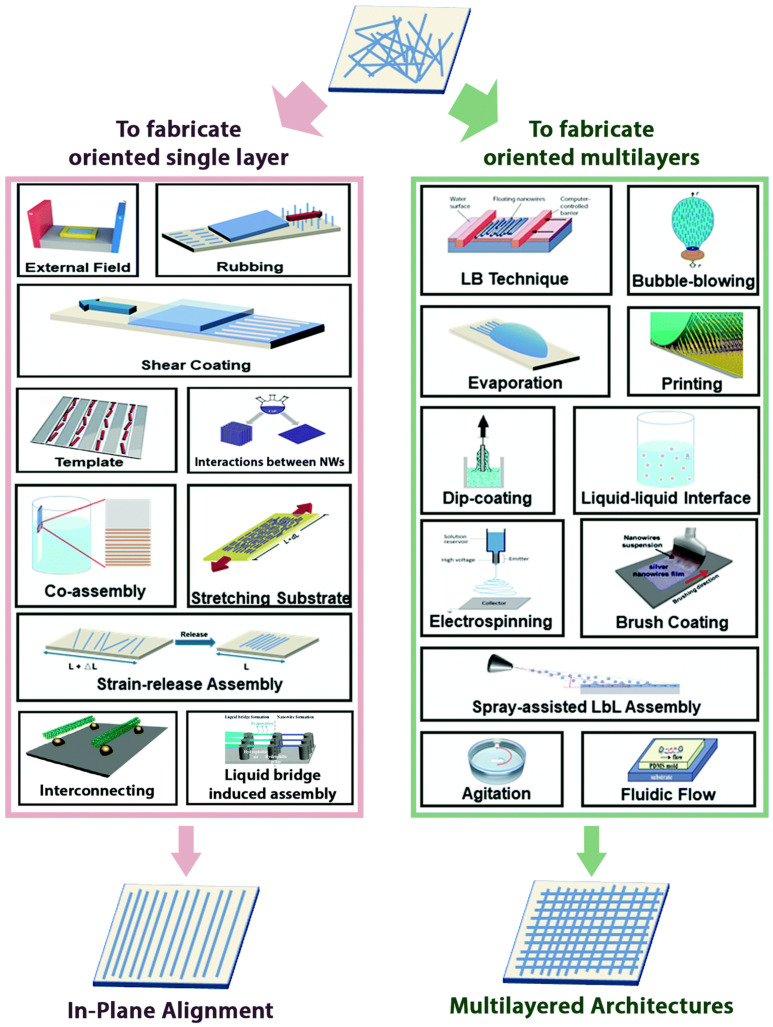
Schematic illustration of assembling methods that can be applied to fabricate in-plane NW arrays and multilayer aligned NWs. Reprinted from reference [54] with permission from the Royal Society of Chemistry.

For sensor fabrication, NWs are usually deposited from a suspension in solution onto the desired substrates. The early used drop-casting technique works well for studying the single NW-based devices, but it can only lead to randomly assembled NWs with poor properties [64,125,126]. The controlled assembly of NWs into the aligned structure is necessary to produce large-scale and high-performance NW-based gas sensors. To date, various assembly strategies have been developed on various substrates. A very recent review paper by Hu et al., comprehensively presents the in-plane aligned assembly of 1D nanoobjects [54]. They summarize the main techniques that lead to oriented NW monolayers or multilayers, including assembly by external fields, shear coating, assembly by template substrates, stretching of the substrate, Langmuir–Blodgett technique, evaporation-induced assembly, contact printing, dip-coating, assembly at liquid–liquid interfaces, layer-by-layer assembly, and so on (Figure 3). These techniques can be categorized based on the driving forces of template, interface, interaction, external field, mechanical force and shear force that governs the oriented assembly. Understanding the advantages and limitations of each method is necessary to choose the best-suited strategy for ordered NW arrays with specific NW density, position, area, architecture and alignment direction.

### 2.3. Electrically Transduced Sensing Architectures

**Figure 4:**
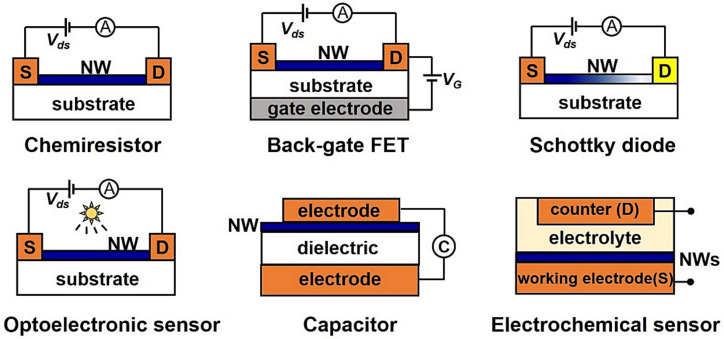
Schematic illustration of electrically transduced gas sensors with semiconducting metal oxide-based nanowires (SMO-NWs) in the horizontal direction. NW, S, D, V*_ds_*, V*_G_*, A, C are short for nanowire, source electrode, drain electrode, bias voltage, gate voltage, ammeter and capacitance meter, respectively.

Most SMO-NWs are arranged in a horizontal direction. The architectures of electrically transduced SMO-NWs gas sensors generally include chemiresistor, FET, diode, optoelectronic sensor, chemical capacitor and electrochemical sensor, as shown in Figure 4. For SMO-NWs in this review, we particularly focus on the general configuration and operation mechanism of chemiresistor and FET.

A chemiresistor consists of two electrodes connected with sensing material, i.e., NWs assembled or as grown on an insulating substrate. The change in resistance or current of the sensor device is measured to analyze the concentration of the target gas. More than half of the reported gas sensors are based on the chemiresistive architecture for their simplicity, compatibility with conventional DC circuits, low cost and high accuracy. Notably, optoelectronic chemiresistors have attracted considerable attention for their enhanced sensing response by creating photo-generated carriers participating in the surface reaction with adsorbed oxygen under light irradiation [32,127]. High-performance, monolithic photoactivated gas sensors based on the integration of gas-sensitive SMO-NWs on micro light-emitting diodes have been achieved by Cho et al., for practical applications in mobile IoT devices [128].

A FET device consists of source and drains electrodes, a sensing material channel, an insulating gate oxide, and a gate electrode. FET sensor is another widely applied device architecture for electronic sensing of gaseous analytes due to its potential for miniaturization, high sensitivity and fast response. This three-electrode transistor architecture offers much more data for sensing analysis (including not just resistance, but mobility, threshold voltage, subthreshold, etc.) so that it can provide more insights into the gas-sensing mechanism. For example, the Liao group designed metal nanoparticle-decorated In_2_O_3_ NW FETs that worked at the deep-enhancement mode to achieve high selectivity [129]. Our previous work has reported the crystal-defect-dependent gas-sensing mechanism of the single ZnO NW FET sensors by comprehensively studying the roles of the surface charge layer and donor and acceptor crystal defects [64]. In addition to gas detection, FET sensor platforms also show tremendous potential for ions and biological sensing.

The Schottky-contacted sensor, formed by a semiconductor with metal/metal-like materials, is an important type of gas sensor based on diodes. According to the Schottky–Mott theory proposed in 1939, the Schottky barrier is generated by an exchange of carriers at the metal–semiconductor interface, and the depletion region generates on the semiconductor side accordingly [130,131]. The Schottky barrier height (SBH) can be modulated via external stimulation like adsorbed gas molecules, causing the current changes exponentially with the SBH under a fixed bias. This explains the high sensitivity in Schottky-contacted sensors. Wei et al., reported that the Pt/ZnO NW Schottky junction achieved a significant improvement in performance with respect to sensing CO, as compared to the performance of the Ohmic contact device [132]. The response and recovery time can be shortened by a factor of 7. Schottky diodes based on the ZnO NWs, SnO_2_ NWs and their heterostructures also showed high sensor response with respect to nitrogen dioxide (NO_2_), hydrogen (H_2_) and ammonia (NH_3_) [132,133,134,135,136,137,138,139]. Several methods to improve the performance of Schottky-contacted gas sensors are also summarized in a recent review by Meng and Li [71].

Other electrically transduced sensor architectures have also been reported. Several electrochemical sensors based on NWs like tungsten oxide, CuO, ZnO and vanadium monoxide can be found in the literature, showing high selectivity and quantitative analytical information to target gases, pH, humidity, glucose and DNA [27,36,49,140,141,142,143,144,145,146]. Capacitive-type sensors define the sensitivity using the change in capacitance at a fixed voltage, and they are not generally applied to detecting inorganic gases [147,148]. SMO-NWs gas sensors based on these architectures are relatively less studied, either because of the complexity in fabrication and measurement or low stability.

Besides being arranged in a horizontal direction, recently, many nanowires have been developed in a vertical architecture [149,150,151,152,153]. Multiple vertically aligned nanowires (VA-NWs) engage in the sensing procedure, and thus the output signal is substantially increased. The VA-NWs also have a higher exposed surface area than horizontal NWs because of the smaller contact area with the substrates. Therefore, the VA-NWs can be used as versatile platforms for gas sensing applications. Typically, Hung et al., reviewed the methods for on-chip growth of vertical SMO-NWs and their sensor performance [84]. Other materials like silicon and GaN are also reported for VA-NWs gas sensors. For instance, Ali et al., fabricated vertically aligned silicon nanowires (VA-Si NWs) by metal-assisted chemical etching. The VA-Si NW gas sensor exhibited a response of 11.5–17.1 to 10–50 ppm H_2_ at 100 °C.

### 2.4. Sensor Performance

The performance of gas sensors is generally performed in a dynamic or static test instrument with the temperature control module, gas flow controllers and data collection system. For practical sensor products, important performance parameters include the sensitivity, the limit of detection (LoD), the selectivity, the response and recovery time, the stability and the power consumption.

Sensitivity and LoD are highly dependent on the physical form, structure and constituent of the sensor material. For SMO-NWs with increased surface area, high sensitivity and low LoD are extensively reported for various NW devices. In most cases, the sensitivity *S* is defined by the ratio of electrical parameters (like sensor resistance) in air and in target gas. The LoD of the particular analyte is related to the noise level of the sensor. According to the International Union of Pure and Applied Chemistry (IUPAC), a reliable LoD can be calculated as a concentration of the analyte, which causes a response 3 times higher than the noise level of the device [154,155]. Due to the negative impacts on both environment and human life in many countries, the Environmental Protection Agency (EPA) has regulated the limit of exposure to toxic and explosive gases, thus driving the continuous development of high-sensitive gas sensors.

Selectivity is defined by the ratios of the sensitivity of the target analyte to that of interfering gases. It is widely known that selectivity is one of the biggest challenges for semiconductor sensors, as their main sensing mechanism is based on the surface reaction of detectants with ionsorbed oxygen. Gas sensors present responses to all oxidizing and reducing gases that contribute to the changed free carrier concentration of the material. To date, most reported SMO-NW sensors obtained enhanced selectivity from functionalization with metal nanoparticles or semiconducting elements that interact selectively with a target gas or form heterojunctions with SMO-NWs [57,80,81,156,157,158]. Recently, several methods have been developed to improve selectivity, such as pulsed temperature modulation, microdiffusion, use of thermal gradients or cycled-temperature regime [159,160,161,162,163]. Selectivity toward specific gases can also be achieved by optimizing the sensor architecture, designing sensor arrays and training machine-learning-based classifiers (see details in Section 5.1) [30,37,145,157,164,165,166,167,168,169,170,171,172,173,174].

Response time is determined by 90% of its final amplitude after analyte exposure, and the recovery time is the time to decrease to 10% of the peak amplitude after removing the analyte. In the reported literature, response time is governed by the factors of surface area, temperature and catalysis [164,175,176,177]. Procedures for promoting sensor recovery mainly include resistive switching, heat treatment and ultraviolet light exposure [178,179,180,181,182,183]. Moreover, long-term stability is also an important parameter for reliable sensors and can be quantified by the response degradation of a device over time. Many reported SMO-NW sensors remained unchanged over several months or more [29,184,185]. The power consumption mainly originates from the electrical resistors for heating, which is the main power-hungry part of the sensor device. In Section 4, we discuss the current sensor technologies to optimize the power budget.

## 3. Structure-Performance Relationship

Understanding the fundamental mechanism is an essential topic of gas sensor research. In the 1980s and early 1990s, significant contributions were made by Yamazoe et al., and Morrison, describing the sensitization mechanism of metal/metal oxide-loaded SMO and the effects of size on gas sensitivity [68,69]. Until today, these mechanisms are the basis for designing gas sensing materials. However, the structure of sensing materials is becoming more complex and diversified, and it is difficult to clearly describe the experimental findings using a single model. For SMO-NWs, the reception and transduction of gas information are related to the changes of surface depletion layer that can be influenced by the size and shape of NWs, the defect engineering, the doped or loaded additives, and the contact geometry. This section intends to clarify the structure-function relationships that are important for the knowledge-based design of high-performance gas sensors.

### 3.1. Size and Shape

**Figure 5:**
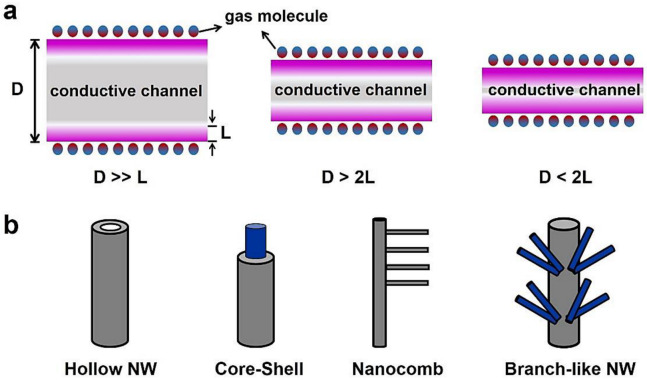
Size and shape-related sensing model. (**a**) Schematic illustration of the relation between the NW diameter (D) and the depth of the surface charge layer (L). (**b**) Various classes of SMO-NWs with porous or branch-like structures.

Size-related gas sensitivity can be explained by the classical D-L model [61], which compares the NW diameter (D) and depth of surface charge layer (L). Large SMO-NWs offer a low surface area-volume ratio, and only the geometrical surface can be affected by the gas molecules. As a result, the changes of L are restricted to a small part of the sensing material, and the electrical transduction can hardly be changed. When D is comparable to or less than 2L, the contribution of changes in surface conductivity becomes prominent, as shown in Figure 5a. This phenomenon has been well observed in many single crystalline NWs, which show enhanced gas sensitivity for a smaller diameter [186,187,188].

Another effective way to achieve high sensitivity is by using porous or branch-like structures, as shown in Figure 5b. The increased surface area facilitates the interactions between the sensitive material and the surrounding gases, which is the origin of enhanced sensor response. For example, hollow SMO-NW (nanotube) has outer as well as inner surfaces. In 2011, Liu and coworkers synthesized aligned ZnO nanotubes through combined electrospinning and sputtering techniques [189]. The sensor response is 3.6 to 100 ppm H_2_, much higher than that of ZnO film. Choi and Chang reported the porous structured ZnO nanotube that showed improved H_2_ sensing response of 4.2 times of porous ZnO film [190]. Other nanotube sensors like TiO_2_, SnO_2_, and ZnO/SnO_2_ composites were also developed for detecting NO_2_, butanone, ethanol, and other gases [140,189,190,191,192,193,194,195,196]. Branch-like NWs can be classified as homogeneous or heterogeneous structure. ZnO nanocomb with the teeth parts is considered as a homogeneous structure. Apart from the greater surface-to-volume ratio, Pan et al., discovered that the teeth part of a ZnO nanocomb could serve as a “negative-potential gate” after accumulating electrons captured by surface adsorbed NO_2_ molecules, which also contributed to the higher NO_2_ sensitivity [124,197]. Solution-based synthesis, especially the hydrothermal method, has been considered a simple and powerful route for the preparation of hierarchical heterojunctions, such as In_2_O_3_/ZnO core-shell NW and TiO_2_/Sn_3_O_4_ brush-like NW [60,81,95,158,198,199,200]. The overall enhanced sensitivity of heterostructures has been generally reported to originate from the electrical contributions of oxide-oxide heterojunction (p-n, p-p or n-n heterojunction) and the spillover effect.

### 3.2. Defect Engineering

**Figure 6:**
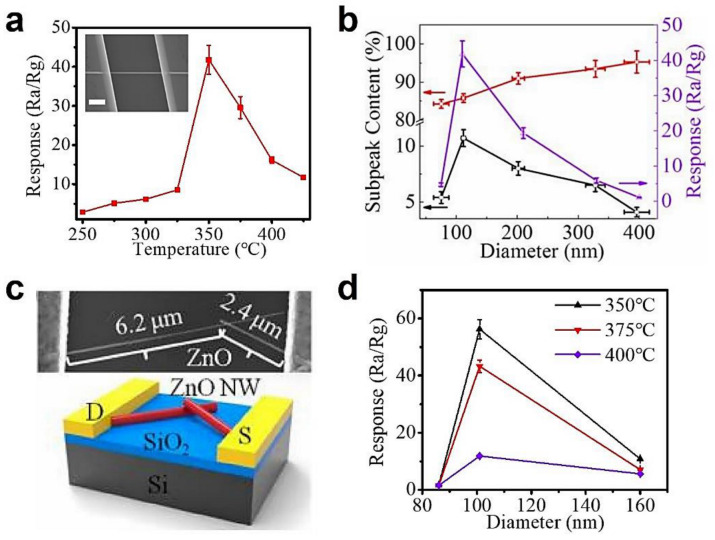
Crystal-defect-dependent gas-sensing mechanism of the single ZnO NW sensors. (**a**) Responses to 5 ppm acetone versus operating temperature of the single ZnO NW with the diameter of ~110 nm. The inset is the corresponding SEM image of the gas sensor. (**b**) Relationship between acetone response of the ZnO NWs at 350 °C (Δ) and the donor level (〇) and acceptor level (⬜) subpeak contents. (**c**) SEM image and schematic of an NW (~110 nm) contact-based device. (**d**) Responses to 5 ppm acetone as a function of the diameter of the contacting ZnO NWs. Reprinted from reference [64] with permission from the American Chemical Society.

Gas-sensing properties of metal oxide materials are strongly related to the crystal defects of electron donor and acceptor [201,202,203,204,205,206,207]. The micro-(μ) photoluminescence (PL) spectroscopy is an effective technique to probe the crystal defects of individual nanostructures. In the case of pure ZnO NWs, the deep level emission of μPL spectra originates from the donor level like oxygen vacancy (V_O_) and zinc interstitial (Zn_i_), as well as acceptor levels like V_Zn_, O_i_, and O_Zn_. The enhanced gas response by defect engineering was mainly attributed to the rich electron donors for absorbing the largest content of oxygen species. For example, Hong et al., reported that the sensitivity to NO_2_ was linearly proportional to the PL intensity of V_O_ in ZnO NWs [201]. Xue et al., reported the formation process of rich V_O_ and Zn_i_ defects by studying the ZnO nanodishes and achieved an excellent response of 49 to 100 ppm ethanol at 230 °C [202]. The crystal facet-dependent gas sensing property proposed by Xu et al., was also attributed to the large amount of V_O_ and dangling bonds existing in ZnO nanosheets with exposed crystal facet (0001) [203]. This mechanism has further been verified by our group, as shown in Figure 6 [64]. According to the μPL subpeaks discriminated by Gaussian deconvolution, the single ZnO NW with a diameter of ~110 nm has the maximum donors and thus displays the best sensor response to acetone at 350 °C (Figure 6a,b). This tendency is minimally affected by the junction in the NW contact-based device (Figure 6c,d). We propose three models of sensing mechanism, including the thinner single crystalline ZnO NW, the medium ZnO NW with many donor-related defects, and the thicker ZnO NW with equal donor- and acceptor-related defects. The intrinsic excitation in the single crystalline ZnO merely generates a few free electrons. While for thicker ZnO NW, many electrons are consumed by the acceptor level. Therefore, only the medium ZnO NWs have enough free electrons to adsorb more oxygen molecules, which contribute to the redox reactions with the target gas.

### 3.3. Doped or Loaded Additives

**Figure 7:**
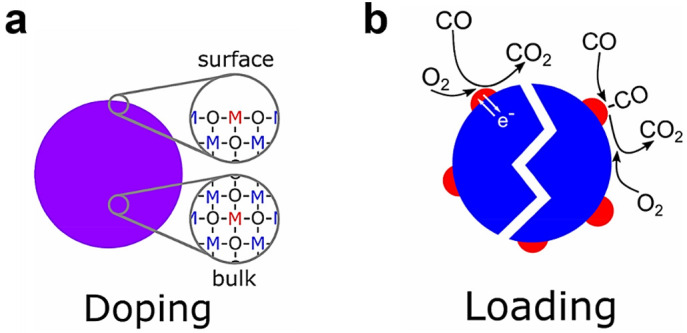
Different structures of SMO (blue) and added metal/metal oxide (red). For doped SMO, additives are incorporated in the SMO lattice at the surface and/or in the bulk (figure (**a**)). For loaded SMO, additives form a separate phase, which is smaller than the actual SMO grain and located at the surface of SMO (figure (**b**)). Reprinted from reference [70] with permission from the American Chemical Society.

Introducing additives in SMO-NWs is the most common way to achieve high sensitivity, selectivity, and stability. The additives are loaded or doped with an additional compound, typically noble, transition metals or other SMO. Doped SMO is the structure where additives are incorporated in the lattice of SMO, as shown in Figure 7. Loaded SMO is a structure where the additive phase is separated from SMO. Both of them can change the chemical and electrical sensing properties of SMO materials.

The chemical contributions to sensor performance are mainly related to oxidation catalysis, the sensing mechanism of which includes: (i) shift the reaction to the additive surface; (ii) activation of lattice oxygen at the SMO−additive interface; and (iii) adsorption and activation of reactive species on the additive surface and a subsequent spillover onto the SMO surface. As an example, the CuO-decorated SnO_2_ NWs showed a high response of 3261—around 400 times that of pure SnO_2_ NW to 2 ppm hydrogen sulfide (H_2_S), because that CuO selectively react with H_2_S, forming CuS [156]. A well-studied example for activation of lattice oxygen is Pt-doped SnO_2_ [208,209,210]. Extended X-ray absorption fine structure (EXAFS) and X-ray diffraction (XRD) analyses showed that Pt^4+^ could be easily incorporated in the SnO_2_ lattice by replacing Sn^4+^, leading to activation of lattice oxygen and an improved activation of methane adsorption [209,211]. In the presence of Pt or Pd catalysts on the SMO-NWs, H_2_ binds, dissociates, and spillover onto the SMO surface, thereby modifying the space charge layers of the supporting SMO. Based on this sensing mechanism, high sensitive and selective H_2_ sensors have been reported by many research groups [79,90,103,212,213,214].

**Figure 8:**
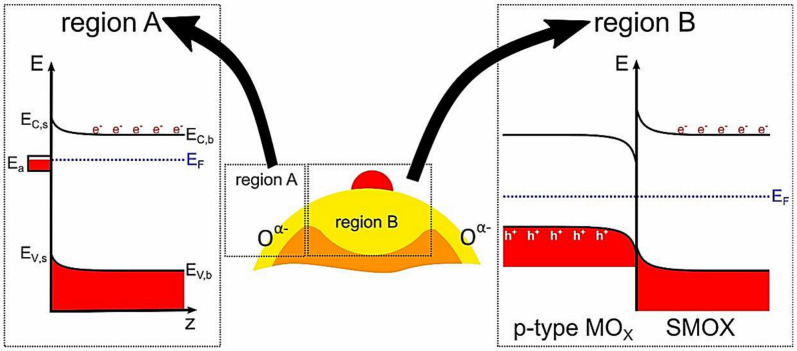
Schematic illustration of the electric contributions. **Region A** corresponds to an unaffected surface, where the space charge layer is controlled by the ionosorption of oxygen, forming a surface state (E_a_). **Region B** corresponds to the heterojunction, where the space charge layer is controlled by the Fermi-level of the loading. Reprinted from reference [70] with permission from the American Chemical Society.

The electrical contributions to sensor performance are related to the band bending by surface states and the electronic coupling in the form of heterojunction between the additive and SMO material (Figure 8). On one hand, the surface band bending occurs in the presence of charge located at the SMO surface or SMO-additive interface. For n-type SMO, the electronic sensitization by an initial upward band bending depends on the energy level and concentration of surface state, as shown in region A of Figure 8 [70]. The relationship of the sensor signal and surface band bending is given by *S* = exp[(*e*V_s_)/(*k*_B_*T*)], where *e* is the elementary charge, vs. is the surface potential, *k*_B_ is the Boltzmann constant, and *T* is the temperature. This initial surface band bending has a big impact on the conduction mechanism and the concentration of surface oxygen species. Rebholz et al., showed the existence of an intrinsic surface band bending for sol–gel-made SnO_2_ materials by simultaneous work function measurements [211]. Moreover, the 1 at % Ni-loaded SnO_2_ was reported to have a huge intrinsic surface band bending and an enhanced sensing performance in comparison to the undoped material. On the other hand, the heterojunction caused by an alignment of two Fermi-levels can lead to a higher upward band bending, as shown in the region B of Figure 8. This effect was observed in many metal/SMO or p-n SMO heterostructures [58,74,158,215,216]. One example is the acetone sensing with Rh-loaded WO_3_ [216]. When exposed to acetone, sensor resistance decreased. Meanwhile, the heterojunction-induced space charge layer got thinner and thus less affected the WO_3_ oxygen species, resulting in more W-O band in the infrared radiation (IR) spectroscopy. Our previous research also demonstrated that the Fermi-level control sensitization is a powerful approach to rationally design and optimize gas sensing materials [58]. Comprehensive reviews on this topic can also be found in the literature [70,217].

### 3.4. Contact Geometry

**Figure 9:**
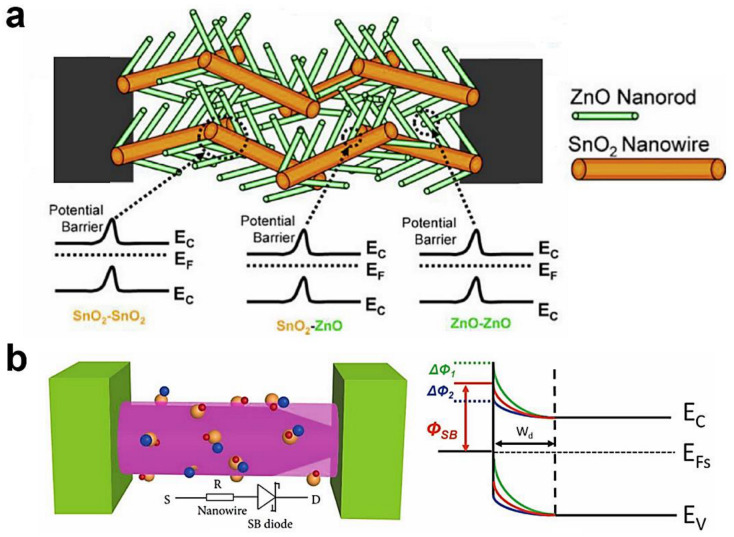
Schematic of NW contact-based sensing mechanisms. (**a**) The schematic of the gas sensing mechanism of NW-NW junctions based on SnO_2_/ZnO hierarchical nanostructures. Reprinted from reference [72] with permission from Elsevier B. V. (**b**) The structure of Schottky-contacted NW sensor, and the change of Schottky barrier height (SBH) and depletion region under the external stimulation. Reprinted from reference [218] with permission from Wiley-VCH.

According to the device structure and working principle, NW contact geometry can be divided into two types: inter-NW junction and Schottky junction, as shown in Figure 9 [71,72,218]. For single NW devices, sensing mechanisms are dependent on the interaction between the analyte and individual NW. While for NW networks, NW-NW junction-type of contacts exist, which are extensively investigated for their ability to control the barrier height and achieve the on-chip fabrication of NW gas sensors [73,93,171,219,220,221,222]. According to the semiconductor nature of materials, NW-NW junctions include n-n, p-p and n-p inter-junctions, as shown in Figure 9a.

Numerous reports available in the literature demonstrate that NW-NW junctions facilitate the low concentration gas detection, as the conduction path involves tunneling through the depletion or accumulation layer. Park et al., reported x^2^ the junction-tuned SnO_2_ NW sensors by changing the spacing of patterned-interdigital electrodes (PIEs) [221]. The narrower spacing of PIEs led to a high density of NW-NW junctions, and thus superior properties for NO_2_ sensing. Cui et al., fabricated highly sensitive H_2_S sensors based on Cu_2_O/Co_3_O_4_ p-p heterojunction and achieved a sub-ppm LoD [215]. The difference between n-n, p-p and n-p junctions was studied based on the air-bridged ZnO/CuO NWs [171]. In ZnO/ZnO n-n junction and CuO/CuO p-p junction, the electron or hole must pass the overlapping NWs over the barrier height. In ZnO/CuO n-p junction devices, electrons flow over the build-in potential, whereas the less effective transduction for p-type CuO decreases the sensitivities of the gases. The interfacial potential energy barrier (*V*) related resistance (*R*) can be defined as *R = R*_0_ exp[(*eV*)/(*k*_B_*T*)]. Apart from this energy barrier, some papers proposed that the inter-NW junction can also narrow the charge conductance channel via the formation of the depletion region, and thus make the resulting depletion region boundary more sensitive than that of the depletion region created directly by the adsorption and desorption of oxygen [223,224,225]. The origin and sensing mechanism of the Schottky junction in Figure 9b have been discussed in Section 2.3. A comprehensive review of the topic of Schottky-contacted NW sensors can also be found in the recent literature [71,134].

## 4. Toward Low Power Consumption

Power consumption is a tough problem both for the application of SMO sensors in IoT programs and the integration of metal oxides into flexible and/or wearable sensors. Strategies for ultra-low power consumption are concluded as thermal isolation, self-heating and developing room-temperature sensors, which work either by decreasing the sensing area or by reducing the working temperature.

### 4.1. Thermal Isolation by MEMS Techniques

**Figure 10:**
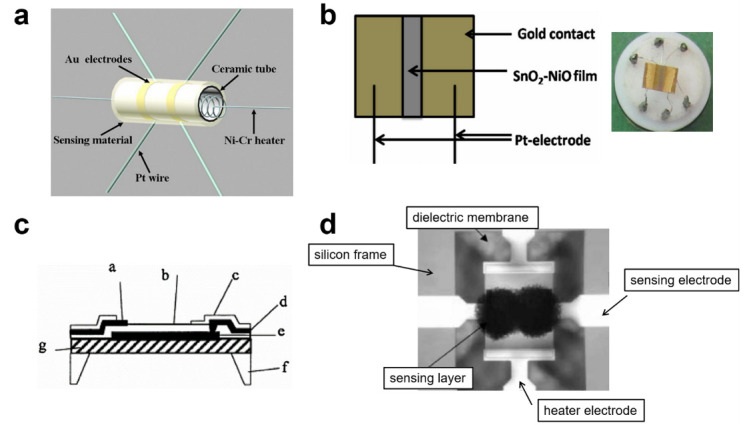
SMO sensors based on various substrates. (**a**) Schematic diagram of the sensor based on the ceramic tube substrate. Reprinted from reference [226] with permission from Elsevier B. V. (**b**) Schematic and photo of the sensor based on alumina plate. Reprinted with permission from [233]. (**c**,**d**) Scheme and photo of the microhotplate fabricated using silicon micro electro mechanical system (MEMS) techniques. The a–g in the figure (**c**) corresponds to the metal line for the sensing layer, dielectric layer, passivation layer, heater metal, polysilicon heater, silicon frame, and dielectric membrane, respectively. Reprinted with permission from [234].

Most of the reported SMO sensors are based on the ceramic tubes in Figure 10a [226,227,228,229,230,231,232]. The sensors are made by coating alumina tube with the solution based SMO paste to form a thin sensing layer. The heating wire of Ni-Cr is inserted in the tube to indirectly heat the sensor. A pair of gold electrodes are installed by twining gold wires on the tube to measure the electrical signal. The power consumption of this type of gas sensor is more than 1 W. Some research tried to decrease the size of ceramic substrates and fabricated SMO sensors on alumina plates (Figure 10b) [233]. The sensing materials are also made into a slurry and then deposited onto the alumina plates with a screen-printed platinum heater. This method helps to reduce the power consumption to hundreds of milliwatts. However, the low manufacturing efficiency, lack of integration capability, together with large sensor-to-sensor variations, limits their practical application and marketing.

The emergence of MEMS techniques has opened up new options for the realization of low energy dissipation, device miniaturization, large-scale device production. The study of MEMS gas sensors started in the early 1990s. The scheme, structure and photo of one first sensor based on this approach were first presented by Chaudret et al., as shown in Figure 10c [234]. The hot sensing area is located at a small scale by etching of silicon from the back sides of the wafer, and in this way, the thermal inertia is drastically reduced. All this period since 90th was dedicated to optimize MEMS layout, to choose optimal materials for the heater and for the insulating layer, and to improve the adhesion between materials at high temperature. Current mass-production silicon MEMS technology has already permitted the effective heating of microhotplates at temperatures lower than approximately 400–450 °C with the power consumption less than 50 mW [77,235,236,237,238]. However, the integration of sensing layers with the MEMS microhotplates is still a technological challenge.

**Figure 11:**
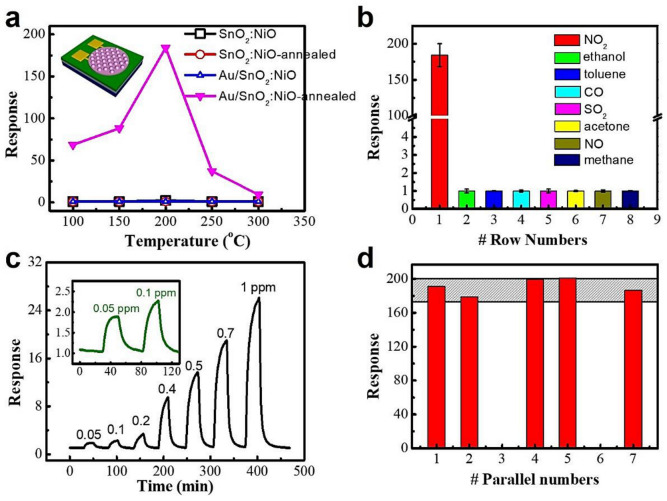
MEMS compatible NO_2_ sensors based on sputtered SnO_2_: NiO thin films on self-assembled Au nanoparticle arrays. (**a**) Response to 5 ppm NO_2_ vs. operating temperature of the SnO_2_: NiO, SnO_2_: NiO/Au and Au/SnO_2_:NiO thin-film sensors. Inset: Schematic diagram of the sensor. (**b**) The selectivity of the Au/SnO_2_: NiO sensor. (**c**) The dynamic sensing response measurements of the Au/SnO_2_: NiO sensor at 200 °C. The inset figure shows the measured LoD is 50 ppb. (**d**) Comparison of responses of the Au/SnO_2_: NiO sensors with different numbers of devices in parallel to 5 ppm NO_2_ at 200 °C. The gray rectangle shows that the device deviation is less than 15%. Reprinted with permission from [58].

Up to now, several novel MEMS gas sensors have been reported, which can be divided into three categories. In the first case, the slurry of chemically synthesized nanomaterials is drop-coated or printed onto the small and suspending active area of MEMS microhotplates [75,122,237,238,239,240,241]. For example, Andio et al., have demonstrated a high-performance CO gas sensor with SnO_2_ nanoparticle-laden ink deposited on a microhotplate via inkjet printing [235]. Li et al., reported the efficient hierarchical mixed Pd/SnO_2_ porous composites deposited on the microheaters using the microdispensing method and achieved high sensing performance towards ethanol at low power consumption [238]. These kinds of methods are particularly difficult and complicated with low yield, low efficiency and large sample-to-sample deviation. For the second category, the site-specific growth of SMO-NWs on microheaters can directly achieve on-chip integration. Hrachowina et al., proved this concept by growing SnO_2_, WO_3_, and Ge NWs on the same microheater chip using CVD techniques [56]. The discrimination between CO, NO_2_, and different levels of relative humidity was obtained by the obtained high-performance gas sensors and the principal component analysis (PCA) method. Nevertheless, the microheater substrates may degrade and even be destroyed under high-temperature conditions of the NWs growth. In the third category, sensing films are deposited by the typical MEMS or MEMS compatible techniques, such as sputtering, evaporation, chemical vapor deposition (CVD) and self-assembly [233,236,242,243,244,245]. Additional thermal oxidation, annealing or acid treatments are subsequently needed to modify the as-deposited compact and amorphous structure. For example, Kang et al. reported a sputtered SnO_2_ thin film-based micro gas sensor, which showed a response of 6–25 ppm toluene at 450 °C [236]. Our group has designed a MEMS compatible gas sensor with sputtered SnO_2_:NiO thin film deposited on self-assembled Au nanoparticle arrays and obtained a high response of ~185 to 5 ppm NO_2_, as shown in Figure 11 [58]. These Au/SnO_2_:NiO sensors exhibit a high response of ~185 to 5 ppm NO_2_, low LoD of 50 ppb, high selectivity, good stability and also sensor deviation of less than 15%. Moreover, we also achieved activating the sputtered SnO_2_:NiO thin film by fabricating cross-linked network structures and by Pd-doping for highly sensitive ethanol detection [29,246]. This type of MEMS sensor shows promising potency in the production of wafer-scale gas sensors with low sensor-to-sensor deviation. Even so, many efforts are still highly desirable towards more simple strategies to control sensing film parameters, including the composition, structure and doping state.

### 4.2. Self-Heating

The self-heating operation is to make use of the local high temperature created by the Joule dissipation during the electrical probing. This strategy is typically proposed for chemiresistor based on monocrystalline NWs [45,59,80,81,82,84,247,248,249,250,251,252,253]. With the bias current applied in conductometric measurements, NWs heat up to relatively high temperatures by the Joule effect and can even melt. The tiny mass of NW allows a remarkable increase in temperature, even when only a small bias voltage is applied. According to the reported self-heated devices based on single SnO_2_ NW, working temperatures in the range of 100–400 °C can be achieved with the power consumptions below 100 μW [59,82,254,255]. In addition to the ultra-low working power, the self-heating approach has three other advantages: (1) making the device architectures more simple by removing the need for external heaters, (2) improving the sensor response with fast thermal response times, and (3) allowing good sensing performances on flexible/wearable substrates. Typically, Meng et al. utilized a pulsed self-Joule-heating of suspended SnO_2_ NW, which enabled not only the gigantic reduction of energy consumption down to 10^2^ pJ/s but also enhancement of the sensitivity for electrical sensing of NO_2_ (100 ppb) [256].

However, fabricating efficient self-heating sensor devices for practical application is still distant. First, the calibration and control of the power–temperature characteristics of NW devices are extremely difficult. Traditional techniques based on the thermal equilibrium between sample and probe are not suitable for NW sensors due to the huge perturbations in the temperature during the measurement. Only estimated values of the temperature can be quickly obtained by indirectly comparing the resistance or gas response in self-heating and in external heating operation. The thermal losses by conduction with the substrates, the contact pads, and the surrounding gas also limit the accuracy in controlling the actual temperature reaching the self-heating mode [257,258,259,260,261,262]. Second, the dimension, shape and constitute of NWs, have a strong impact on the thermal distribution, electrical properties, as well as thermal conductivity. As a reference, the temperature is inversely proportional to the square of the radius of NW [82]. Thus, the synthesis of monodispersedly sized SMO-NWs is crucial for the large-scale production of reliable self-heating sensors. Third, there exists a risk of damaging the sensor due to the burning of the NW at a high input power. While for low input power, the conductivity range of NW sensors requires more complex and expensive electronics. Multiple-wire configurations like aligned NWs and NW networks may help to handle this contradiction and also facilitate sensor integration [73,157,189,263,264]. The recent study of self-heated SMO-NW sensor is reported by Ngoc et al., who present an effective SnO_2_ NW sensor for reducing gases by using the Joule heating effect at inter-NW junctions [59]. The sensor’s power consumption is around 4 mW, already meeting the requirement for application in mobile phones.

### 4.3. Room-Temperature Sensing

Room-temperature sensing has been proposed toward zero power consumption. Nitrogen oxide and hydrogen interaction with SMO nanostructures at room temperature are the most studied areas. The Zhou group reported the detection of NO_2_ down to ppb levels using single and multiple In_2_O_3_ NW FET sensors operating at room temperature [265]. Fan et al., also reported a ZnO NW FET sensor, showing highly sensitive properties toward NO_2_ [266]. A number of articles present that the Pd/SMO or Pt/SMO nanostructures possess the unique sensing mechanism of forming hydride and thus exhibit highly selective H_2_ detection [79,103,213,214]. Due to the slow interaction dynamics, the response speed for room-temperature sensing was relatively low, and extra electro-desorption or optical-desorption was explored to speed up the recovery process and to achieve the complete recovery of measured electronic parameters [178,179,180,181,182,183]. Moreover, the electric field-assisted and light-activated absorption/desorption of target gases were also used to improve the sensor sensitivity and selectivity. Recently, Zhou et al., have prepared a ZnO NW network sensor and studied the room-temperature sensing performance toward trace NO_2_ under ultraviolet (UV) illumination [267]. With UV exposure, the adsorbed oxygen ions were weakly bonded on the ZnO surface, facilitating the reversible response of 157 to 50 ppb NO_2_ at room temperature. Visible light-activated excellent NO_2_ sensors based on ZnO/g-C_3_N_4_ composites are reported, showing the LoD of 38 ppb [268].

Other effective strategies to lower the working temperature are surface modification, additive doping, or fabrication of heterostructures [269,270,271,272,273]. Oleg et al., have reported the sensor devices based on multiple networked Au/ZnO NWs, showing a high response of 40 to 100 ppm H_2_ and a low theoretical LoD below 1 ppm [263]. The high gas response was explained based on a gas sensing mechanism, which includes the modulation of multiple potential barriers between the NWs and the role of noble metal nanoclusters. Pham et al. studied the VLS catalytic growth of the biaxial p-SnO/n-ZnO heterostructured NWs and achieved a good sensing performance to ppb-level NO_2_ at room temperature without light illumination [274]. This p-SnO/n-ZnO device also showed a low LoD of 50 ppb under various relative humidity. Shen et al., reported interesting results for room-temperature sensing performances of mesoporous In_2_O_3_ nanorod arrays on a porous ceramic substrate for ppb-level NO_2_ detection [37]. The response value to 800 ppb NO_2_ was 14.9 with a short response time of 14 s. Non-stoichiometric SMO materials like W_18_O_49_ NW networks showed highly enhanced ammonia sensing properties at room temperature due to the large amounts of oxygen vacancies that facilitate the chemisorption of oxygen at a low temperature [275].

**Figure 12:**
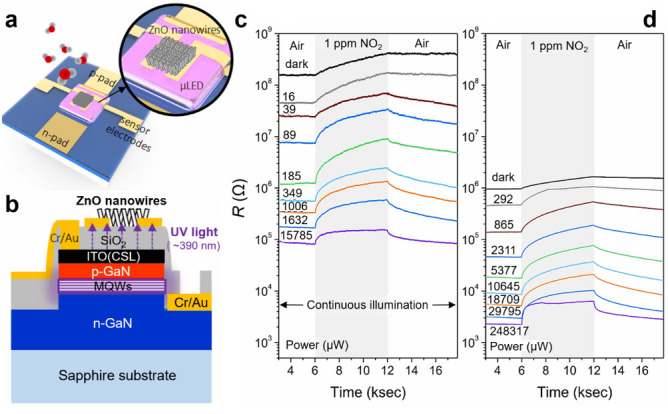
(**a**) Monolithic photoactivated NO_2_ sensor based on the integration of ZnO NWs on μLEDs. Schematic illustration of the top view and (**b**) cross-sectional view of the sensor structure. Responses of monolithic photoactivated gas sensors on 30 × 30 μLED platforms (**c**) and 200 × 200 μLED platforms (**d**) to 1 ppm NO_2_ gas under different input electrical powers at room temperature. Reprinted with permission from [128].

Nowadays, the on-chip fabrication of optoelectronic chemiresistors is attractive for room-temperature monitoring due to their highly enhanced selectivity and easy implementation with micro light-emitting diodes (μLEDs) or optical fiber technology [32,78,127,276,277,278]. Cho et al., designed the monolithic photoactivated gas sensor based on the integration of ZnO NWs on μLEDs, as shown in Figure 12a,b [128]. The sensor showed excellent NO_2_ sensitivity (Δ*R*/*R*_0_ = 605% to 1 ppm NO_2_) at the operating power of 184 μW (Figure 12a,b). Chen et al., also reported the mesostructured ZnO NW photoelectronic formaldehyde sensors that exhibited an LoD of as low as 5 ppb and a response of 1223% (at 10 ppm) [278]. Microlight plate configuration allows for the device miniaturization and the power consumption as low as 30 μW. A comprehensive review on UV-LED photo-activated chemical gas sensors was presented by Espid and Taghipour, illustrating the recent progress in this area [181]. In contrast, there are still challenges to fabricate reliable room-temperature sensor for commercialization. The influence of humidity is a major problem to be solved.

## 5. Sensing Applications toward IoT

Advances in sensing gaseous markers of SMO-NWs illustrate their ability for various applications, such as disease diagnosis by breath sensors, environmental protection, human safety monitoring, as well as novel flexible/wearable electronics. In the last two decades, extensive efforts and exciting scientific discoveries have been reported toward IoT-based projects [10,11,12,13,85,169,279,280,281,282,283,284]. With a better understanding of the two fundamental processes of gas reception and electrical transduction, more high-performance and reliable SMO-NW sensors will be translated into actual production.

### 5.1. Breath Sensor for Disease Diagnosis

Chemoresistive sensors are quite attractive for their promising application in the early diagnosis of chronic diseases and monitoring of physical conditions in a non-invasive, simple, and low-cost way. Due to the abnormal metabolism, human breath contains N_2_, O_2_, water, CO_2_, inert gases, and hundreds of other VOCs typically occurring at very low concentrations (ranging from ppb to ppm level) [285,286,287]. These breath gases can act as the biomarker species, which are generally identified using traditional chromatography−mass spectrometry (GC–MS) with benchtop sensitivity and selectivity [5,288]. Till now, several biomarker species have been studied sufficiently and tested in clinical tests, such as the ethanol tests employed by law enforcement for safe driving [289], the CO_2_ monitoring in intensive care and anesthesia [290], and NO to detect asthma [291]. However, the GC–MS techniques are extremely expensive, time-consuming, complex-to-use, non-portable, and not compatible with on-chip integration. Implementation of SMO-NWs for breath sensing is effective for high sensitivity, chip-scale miniaturization and low-power operation.

An early study for breath sensor was proposed for testing blood alcohol in 1983 when Watson performed the sensing action using a semiconductor sensor of Figaro TGS 812 [292]. With the nanoscale engineering of SMO sensing materials, high sensitivity and lower LoD can be obtained. Recently, several review articles have already been published that provide deep insights into the SMO breath sensors. Alizadeh et al., present a comprehensive review of breath acetone sensors to detect diabetes [18]. They focus on various SMO materials such as WO_3_, ZnO, SnO_2_, NiO, TiO_2_, and different sensor technologies. Tai et al., summarize the latest research on wearable humidity-enabled breathing behaviors monitoring [293]. Nasiri et al., focus on the nanodimensional design of current state-of-the-art breath sensors [294]. Liu et al., review the flexible and stretchable breath sensors, including the sensing materials, sensing mechanisms, and their fabrication methods [295]. Tricoli et al., report the latest achievements in point-of-care monitoring of chronic kidney diseases by sensing nitrogen biomarkers (NH_3_, creatinine and urea) [296]. Moreover, Guntner et al., emphasize the key challenges that currently impede the realization of breath sensors, which include improving the selectivity and stability of breath sensors, understanding the underlying biochemical and physiological mechanisms of breath markers, and acquiring sufficient clinical testing data [280].

**Figure 13:**
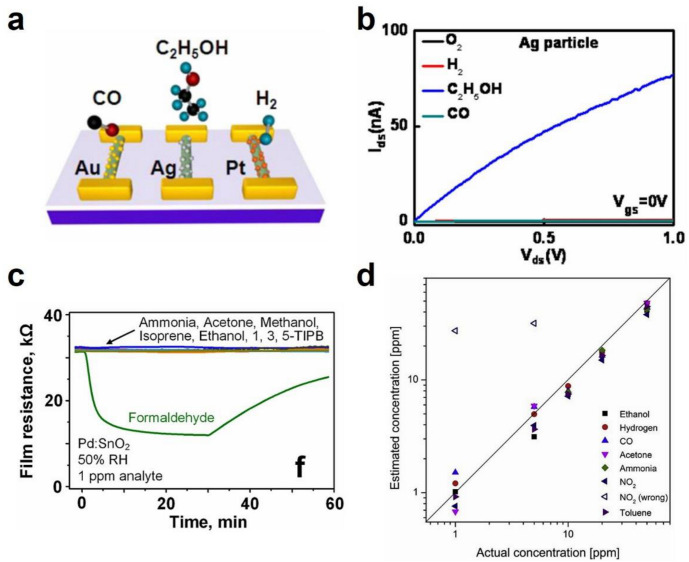
Tailoring selectivity for breath sensor. Schematic (**a**) and gas specific detection toward 100 ppm ethanol (**b**) of the metal nanoparticle-decorated Mg/In_2_O_3_ NW field-effect transistor (FET) sensor. Reprinted with permission from [129]. (**c**) The selective formaldehyde sensing by applying a microporous zeolite membrane above a Pd-doped SnO_2_ sensor. Reprinted with permission from [297]. (**d**) Quantitative prediction from the single SnO_2_ NW sensor by machine learning algorithms. Reprinted with permission from [30].

Effective strategies have been developed for improving the sensor selectivity, including functionalizing with additives, designing sensor arrays, using filters like size-selective membranes, and combining the machine-learning classifiers [166,281,283,294,297,298,299]. As a typical example, Zou et al., used Mg-doped In_2_O_3_ NW FET sensor arrays decorated with Au, Ag, and Pt nanoparticles for the selective detection of CO, C_2_H_5_OH, and H_2_, respectively (Figure 13a) [129]. Figure 13b shows the current–voltage curve of Ag-decorated Mg/In_2_O_3_ NW FET with a response of ~10^3^ to 100 ppm ethanol. Moreover, the selective detection of formaldehyde was reported by combing the zeolite Mobile-Five (MFI) membrane with a Pd-doped SnO_2_ sensor (Figure 13c) [297]. The microporous MFI membrane helped to filter other analytes with large molecules, leading to an effective size cutoff effect for selective gas sensing from complex gas mixtures. Tonezzer achieved the selective sensing and quantitative prediction of VOCs based on a single SnO_2_ NW chemiresistor by applying machine learning algorithms (Figure 13d) [30]. Under a temperature gradient, five signals can be extracted, forming the thermal fingerprint of each specific gas. This system can recognize different gases with an accuracy of 94.3%. In contrast, most VOCs and their breath phenotyping are still to be explored.

### 5.2. Gas Sensor for Environment Engineering

Monitoring air pollutants, especially toxic gases, are driven by regulation. Representative values are published by the State Environmental Protection Administration of the People’s Republic of China, the United States Environmental Protection Agency, the Occupational and Safety Health Administration (OSHA), and the European Union Agency for Safety and Health at Work [300,301,302,303]. In this section, we provide overviews of important gaseous analytes, including earlier and the most recent developments. Particularly, SMO-NW sensors are designed to detect indoor gases like CO, formaldehyde, benzene series (BTEX), and outdoor gases like NH_3_, NO_x_, sulfur dioxide (SO_2_), CO_2_, H_2_S, ozone (O_3_), VOCs, etc.

#### 5.2.1. Detecting Indoor Gases

Indoor pollutions are produced mainly from the incomplete combustion of carbon fuels, natural gas manufacturing, and the production of textiles, resin, wood composites and household materials. Typically, the higher affinity of CO over O_2_ toward the iron porphyrin complexes in hemoglobin causes CO poisoning. Hernandez et al., demonstrated highly sensitive and stable CO sensors using a single SnO_2_ NW [304]. The detection threshold was smaller than 5 ppm, and measurement instability was lower than 4%. The room-temperature CO sensing was achieved by gold nanoparticle (AuNP)-functionalized In_2_O_3_ NW FET [38,129]. The presence of AuNPs helped to enhance the CO oxidation, and this contributed to the high response to a low concentration of CO (200 ppb-5 ppm).

Formaldehyde and BTEX have been classified as a human carcinogen, as they cause nasopharyngeal cancer, pulmonary damage and leukemia. Various SMO-NW sensors have been designed for detecting indoor formaldehyde and BTEX, such as Fe-doped ZnO, NiO-loaded ZnO, CuO/SnO_2_ and SnO_2_/ZnO [74,85,142,158,305,306]. Toluene- and benzene-selective gas sensors were reported based on Pt/Pd functionalized ZnO NWs, alpha-Fe_2_O_3_/SnO_2_ NW arrays, Pd decorated In_2_O_3_, Pd functionalized SnO_2_/ZnO core-shell NWs, Au/ZnO NWs, and other NW materials [80,198,307,308,309]. However, the LoD of most reported formaldehyde and BTEX sensors were above 1 ppm, much higher than the regulated limits of many environmental agencies (60 ppb for formaldehyde and 48 ppb for toluene, according to the files of GB/T 15,516 and GB 14,677 in China).

#### 5.2.2. Detecting Outdoor Gases

Outdoor air pollutions are originated from agricultural processes, automobile exhausts, photochemical reactions, chemical production, as well as bacterial decomposition of animal and human waste. Typically, SO_2_ is highly corrosive and readily oxidized in air to create sulfuric acid. Khan et al., reported a scalable SMO-functionalized GaN NW for precise SO_2_ detection [92,310]. Liu et al., fabricated high-efficiency SnO_2_ sensors with Au nanoparticle-modified SnO_2_, exhibiting a low LoD of 500 ppb and a fast response/recovery (34/14 s) at 200 °C [207]. Other SO_2_ sensors were also reported based on the Mg^2+^ conducting solid electrolyte, TiO_2_ nanotube arrays, Ru/Al_2_O_3_/ZnO composites, Ag-loaded WO_3_, etc. [34,42,180,311,312,313].

Additionally, other representative outdoor gases are monitored. H_2_S at low concentration is very harmful to human eyes, noses and throats. Extensive research developments for H_2_S detection have been reported based on SMO-NWs like aligned CuO NW array, SnO_2_, Au/WO_3_, Cu-doped SnO_2_, CuO/SnO_2_, Mo-doped ZnO NW network, etc [33,62,81,156,157,215,314,315]. Exposures of NH_3_ threaten the eyes, skin, and respiratory system. High-performance NH_3_ sensors have been achieved by WO_3_/W_18_O_49_ heterostructured NWs, self-assembled ZnO NWs, ZnO NW/reduced graphene oxide (rGO) hybrids, SnO_2_ NW, WO_3_ NW, rGO/WO_3_ NW, etc. [316,317,318,319,320,321]. Moreover, CO_2_ is a major public concern in environmental engineering due to its role in global greenhouse warming. The reported CO_2_ detection was achieved by SMO like Al- and Cu-doped ZnO NWs, bismuth oxide (Bi_2_O_3_), Pt/NiO, ZnO, and other sensing materials like LaFeO_3_ and carbon nanotubes [39,237,322,323,324,325,326]. NO_x_ (like NO_2_, NO) from the combustion of chemical plants and motor vehicles can cause irritation in the human respiratory system and contribute to the development of asthma. To avoid repetition, the interested readers are referred to the discussions of NO_2_ sensing in low power consumption sensors (Section 4) and VOC sensing in breath sensors (Section 5.1).

In particular, the photochemical-induced O_3_ that is widely used in purification has been classified as toxic gas as it is hazardous to human health. Current warning levels of O_3_ are ranging from 50 to 100 ppb for different countries. Silva et al., fabricated the O_3_ sensor based on α-Ag_2_WO_4_ nanorod-like structures [327]. The sensor exhibited good sensitivity and a short response (6–7 s) and recovery times (13–16 s) with O_3_ concentrations from 80 to 930 ppb. An In_2_O_3_-based O_3_ sensor prepared by Epifani’s group showed a low LoD of 60 ppb, approaching the regulated values [328]. Other SMO sensing materials for O_3_ detection include In_2_O_3_, SnO_2_, NiO, WO_3_, Au/TiO_2_, Ga_2_O_3_, MnO_2_, CuAlO_2_ [40,76,329,330,331,332,333,334,335,336,337,338,339]. The delafossite-type oxide CuCrO_2_ nanocrystals were considered as a promising material for O_3_ sensing due to the exhibited reversible high-sensitive response at room temperature [339].

### 5.3. Gas Sensors for Personal Safety and Food Safety

#### 5.3.1. Monitoring Gases for Personal Safety

Flammable and combustible gases like methane (CH_4_) and H_2_ pose a threat as they can explode even at low concentrations in the air. The explosive limits of CH_4_ and H_2_ gas concentration are 5% and 4%, respectively [340]. Thus, the detection of CH_4_ and H_2_ is essential for protection and warning purposes in the systems of mines, petroleum fractional distillation plants, chemical factories, and also research laboratories. Additionally, methane is the main component of natural gas. Methane sensor systems for homes are required to prevent the loss of human life and national revenue.

**Figure 14:**
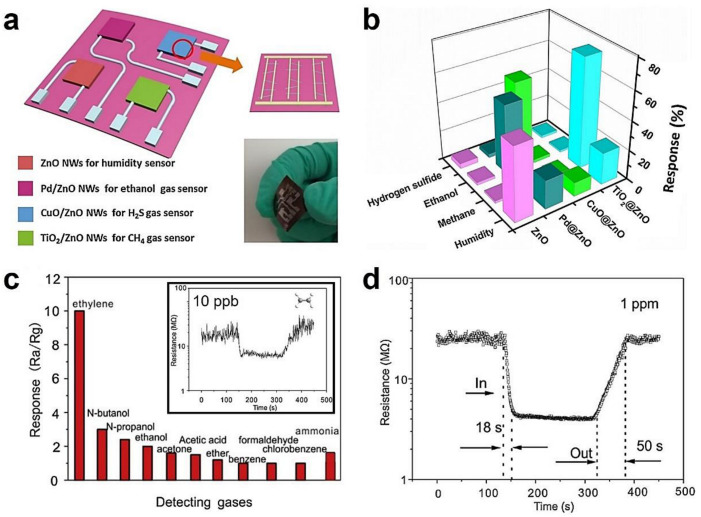
Gas sensors for personal safety and food safety. Schematic, photo (**a**) and selectivity (**b**) of the four different ZnO NW sensing units for detecting humidity, ethanol, H_2_S and CH_4_ at room temperature. Reprinted with permission from [342]. The selectivity, limit of detection (LoD) (**c**) and response–recovery time (**d**) of Pd/rGO/α-Fe_2_O_3_ to 1 ppm ethylene at 250 °C. Reprinted with permission from [343].

SMO-NW-based sensors for both CH_4_ and H_2_ are very common with extensive research. For example, Ni_2_O_3_ decoration of In_2_O_3_ nanostructures has been synthesized for enhanced CH_4_ sensing, where the Ni_2_O_3_ nanoparticles played a catalytic role in methane gas reactions and led to a reduction of operation temperature [341]. Porous Au-embedded WO_3_ NW structure reported by Vuong et al., also showed high response performance toward CH_4_ [33]. Moreover, the real-time monitoring of the mining environment has been reported by designing a flexible self-powered smelling electronic-skin (e-skin) shown in Figure 14a [342]. Figure 14b shows that the e-skin can cross-reactively detect relative humidity (RH), ethanol, H_2_S and CH_4_ (based on RH: bare ZnO NWs, ethanol: Pd/ZnO NWs, H_2_S: TiO_2_/ZnO NWs, and CH_4_: TiO_2_/ZnO NWs), due to the piezoelectric-gas sensing coupling effect. High sensitive H_2_ sensors are often related to Pd-functionalization of SMO nanostructures, the fundamental mechanism of which has been referred to in the doped/loaded additives of SMO (Section 3.3). Beyond that, the Pd electrode contacted SMO-NW sensors have also been reported for H_2_ detection. Kumar et al., designed the H_2_ sensor based on Pd contacted ZnO nanorods [213]. The relative response was 38.7% to 7 ppm H_2_.

#### 5.3.2. Monitoring Gases for Food Safety

Ensuring food safety is of great societal concern. Gas sensors based on SMO-NWs can be used to detect toxins and specific VOCs that are produced in food products during the food aging and transportation processes so that we can avoid food-related illness and minimize food loss. Climacteric fruits like apples and bananas produce plant hormone ethylene at the onset of ripening. Monitoring ethylene concentration is useful in determining the optimal harvesting time and preservation of freshness in storage [344,345,346,347]. Baik et al., reported an electronic nose (e-nose) strategy based on SnO_2_ NW arrays [6]. The selective detection of ethylene was achieved by decorating Ag-nanoparticles that played a catalytic role in selectively sensing. Despite the nonpolarity and low reactivity of ethylene, sub-ppm-level detection has been achieved in recent years. The Liu group demonstrated the Pd nanoparticles and rGO-modified α-Fe_2_O_3_ ethylene sensors that exhibit a low LoD of 10 ppb and a fast response/recovery speed (18 s and 25 s) at 250 °C (Figure 14c,d) [343]. This breakthrough in LoD was contributed to the high specific surface area of hierarchical structure, the catalysis of Pd, and chemically active defect sites of rGO. Jeong et al., have also designed the Cr_2_O_3_/SnO_2_ sensor with a low LoD of 24 ppb for ultra-selectivity and highly sensitive ethylene detection at different humidity conditions [348]. The potential of the Cr_2_O_3_/SnO_2_ sensor for real-time fruit freshness monitoring was demonstrated by using a sensing module wirelessly connected to a smartphone.

Moreover, trimethylamine (TMA) is another gaseous compound that is formed naturally due to the biodegradation of fish and animal products. The freshness of fish and other seafood can be detected by TMA sensors based on SMO materials, such as ZnO, MnO_3_, NiGa_2_O_4_, Au-decorated WO_3_, In_2_O_3_/SnO_2_, TiO_2_, RuO_2_/LaFeO_3_, α-Fe_2_O_3_, etc. [55,176,224,349,350,351,352,353,354,355,356,357,358,359]. As a case in point, Lou et al., fabricated a novel TMA sensor based on the α-Fe_2_O_3_ nanorods/TiO_2_ nanofibers hierarchical heterostructure synthesized by combined electrospinning and hydrothermal technique [360]. The sensor provided excellent sensing performances with the response of 13.9 to 50 ppm TMA gas and fast response/recovery rate (0.5 s/1.5 s), mainly due to the sensing mechanism of NW-NW junctions (Section 3.4). Other target gases for monitoring food safety will not be repeatedly discussed in this section, such as NH_3_ that is responsible for fishy odors, H_2_S that is related to the overcooked and rotten food of eggs, meat and milk, and ethanol that is formed during the decay of fruits and vegetables. Nowadays, the artificial neural network with selected feature data sets is developed for data validation of e-nose, facilitating the decision for effective monitoring of current food conditions [283,361].

### 5.4. Flexible/Wearable Sensors

Flexible/wearable sensors can play a pivotal role in IoT in healthcare [7,282,295,362,363,364,365,366]. However, the SMO-NW-based flexible/wearable sensors are not very common due to the key issues for reliable devices, such as the effects of repeated bending stress, the difficulty to direct grow NWs on flexible substrates, and the low tolerance of sensor devices to high temperature (for plastics: 100–200 °C).

#### 5.4.1. Flexible Gas Sensors

The integration of semiconducting NW arrays on flexible substrates was reported earlier for chemical sensors in 2007 [367]. Heath and his coworkers developed the superlattice nanowire pattern transfer (SNAP) approach to fabricate flexible silicon NW sensors with ppb-level sensitivity to NO_2_. Since then, extensive research has been focused on the integration strategy and device performance of this type of sensor. In 2010, Ahn et al., reported the integration of ZnO nanorods by thermolysis-assisted chemical solution on polyimide substrates [264]. These flexible sensors exhibited enhanced sensing performances with a sensitivity of 3.11 to 100 ppm ethanol and a response/recovery time of 3–5 min at an operating temperature of 300 °C. These results were competitive to that of ZnO nanorod sensors fabricated on a hard SiO_2_ substrate.

**Figure 15:**
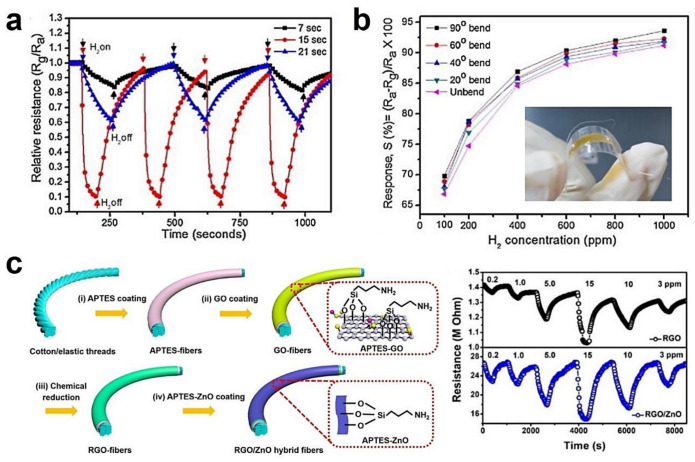
Flexible/wearable SMO gas sensors. Response repeatability of ZnO nanorod sensors at 1000 ppm H_2_ for various Pd-loading conditions at room temperature (**a**), and performance of the flexible sensor for various bending angles (**b**). Reprinted with permission from [212]. Schematic diagram of the fabrication process for rGO/ZnO hybrid fibers and the sensing behaviors of rGO and rGO/ZnO sensors at room temperature (**c**). Reprinted with permission from [86].

Moreover, the low-power sensor strategies like self-heating and room-temperature sensing techniques are very favorable for flexible sensors. Rashid et al., reported a flexible H_2_ sensor based on Pd-decorated ZnO nanorods directly grown on a polyimide tape substrate [212]. As shown in Figure 15a, a response of 91% with good repeatability and stability was achieved for 1000 ppm hydrogen at room temperature, and the sensor LoD was 0.2 ppm. In addition, the most important improvement was that the sensor performance did not significantly degrade, even after bending 10^5^ cycles to an angle of 90° (Figure 15b). This confirmed the mechanical stability of such gas sensors in practical applications.

Self-powered devices based on the concept of piezoelectric nanogenerators (NGs, proposed by Wang et al., [368]) are also suitable for flexible sensors. Research on this topic can be found in the literature [41,342,365,369,370,371]. Uddin et al., demonstrated the low-temperature and flexible self-powered active acetylene (C_2_H_2_) gas sensors based on Ag/ZnO NWs [372]. The piezo-plasmonic effect originated from the piezoelectric output, and optical excitation was the fundamental mechanism that facilitated the enhanced sensitivity of 51.7% to 1000 ppm C_2_H_2_ at room temperature. In contrast, the main challenge in the field of flexible NW sensors is the stability over long-term operation of bending and stretching. Great efforts are still needed to devote to this key issue essential for the validation of the final devices.

#### 5.4.2. Smart Textiles

Smart textiles for wearable sensors are currently attracting more attention for healthcare, military and environmental applications. Although the smart textiles research started more than 20 years ago [366], an early study of textile gas sensors based on SMO fibers was reported by Lim et al., in 2010 [373]. They used the uniform and high-crystalline ZnO nanorods on fabric to fabricate ready-to-wear multifunctional gas sensors. With the coating of a sputtered Pt layer (5 nm), the ZnO-on-cloth exhibited a response of 68% to 500 ppm H_2_ with a response/recovery time of several minutes. Furthermore, the morphology and electrical characteristics of this textile device were found to be robust against mechanical handling, including stretching, twisting and washing of the fabric.

In contrast, most of the reported textile devices are based on conductive polymers (CPs) instead of SMO fibers, mainly because of the poor sensitivity and selectivity of SMO at room temperature [374,375,376,377,378]. Designing rGO-modified SMO fiber is an effective way to solve this problem. Recently, Li et al., have built the textile gas sensor using commercially available cotton/elastic threads as templates and rGO/ZnO as sensing layers, as shown in Figure 15c [86]. The as-prepared conducting fibers showed a highly selective response of 44% to 15 ppm NO_2_, excellent long-term stability over 84 days, a low theoretical LoD of 43.5 ppb, good washing durability, and great mechanical deformation tolerance of bending (3000 cycles), twisting (1000 cycles), and stretching (65% strength). They also present the scalable application of such textiles in weaving multisensor array networks integrated into clothes, showing a great prospect for commercial products. Moreover, they reported another rGO/ZnO textile-based gas sensor, which showed enhanced sensitivity and selectivity to NO_2_ by UV light irradiation [180]. Nowadays, the research on SMO-NW integration into a smart textile is still at the nascent stage. Great challenges exist in the low-power operation, selectivity, and mechanical stability under bending stress and washing treatment.

## 6. Conclusions and Perspectives

The electrical-transduced SMO-NW gas sensors are important potential candidates for the realization of IoT-related sensing technologies due to their intrinsically high sensitivity of NWs and the simplicity and portability of sensor devices. In this review, we have presented the developed synthesizing and assembling strategies for SMO-NWs, the structure-performance relationships that clarify the fundamental sensing mechanisms, the low-power operations, and novel applications toward IoT. Several conclusions could be made with the proposal for future trends.

First, many synthetic routes for SMO-NWs have already been found, which are divided into the top–down and bottom–up strategies. The structural, material and interface designs of SMO-NWs can be obtained by varying growth conditions. Generally, amorphous NW arrays are fabricated in wafer-scale scale by top–down techniques, and high-crystalline NWs are synthesized through the vapor phase or liquid phase process. However, there is still a lack of strategies for monodispersedly sized NWs with high crystallinity. The size distribution-induced averaging effect of NW networks or films may lead to significant performance degradation, and this is a big bottleneck to overcome.

Second, the in-plane alignment of NWs can be achieved by various assembling methods with the driving forces of templates, interfaces, mechanical force, interactions between NWs and external fields. Thus, great improvements have been made for NW arrays with well-defined interspacing and location. Gas sensors based on SMO-NW arrays or networks have been reported for on-chip integration, and yet, the reproducibility, reliability, and consistency of sensing devices have been hardly investigated. Moreover, it is extremely difficult to integrate NWs on MEMS microhotplates because of the small suspending heating area and the poor adhesion between microheater and sensing nanomaterials. Alternative techniques like electrohydrodynamic (EHD) inkjet printing need to be further explored [79,185,378].

Third, most SMO-NWs with additive-functionalization, heterojunctions, hierarchical structures and Schottky-contacts are reported to exhibit high sensing performance. The development of in situ and operando methods enables the clarification of relationships between the NWS’ structure and gas sensing performance. Basic models such as the D-L theory, spillover effect and band bending have already been proposed for understanding the fundamental mechanisms of SMO-NW sensors. However, it is still difficult to quantitatively evaluate the contribution of each effect when dealing with complex or hybrid structures. The knowledge-based rational design of SMO-NW sensors is far from being realized.

Fourth, low-power operation is a critically important issue for commercialization. Great progress has been made to reduce the power consumption of SMO-NW gas sensors, which are summarized as thermal isolation by MEMS techniques, self-heating and room-temperature sensor technologies. In contrast, MEMS compatible sensing films of SMO-NWs are still lacking. The control and efficient design of self-heating sensor devices are far from productive demand. The selectivity and the influence of humidity remain unresolved for most room-temperature gas sensors.

Finally, the innovations of SMO-NW gas sensors have an impact on new relevant areas such as disease diagnosis by breath sensors, environment, personal safety and foods. The development of big data and cloud computing paves the way for sensor-based IoT projects, bringing significant changes in our daily life. Nevertheless, only a few sensor systems are currently commercialized. As for breath sensors, challenges exist in the selective detection of trace target breath markers from more than 800 VOCs species. The emerging machine learning algorithm provides a promising toolbox to construct high-performance sensors (design of sensing materials, device architecture, arrays) and to achieve selective sensing by choosing rational classifiers. The future work should also focus on the integration of NWs on flexible/wearable substrates and the device stability over long-term and harsh bending tests.
